# Incidence and Prognosis of Colorectal Cancer After Heart Transplantation: Data From the Spanish Post-Heart Transplant Tumor Registry

**DOI:** 10.3389/ti.2023.11042

**Published:** 2023-05-19

**Authors:** Marta Sagastagoitia-Fornie, Laura Morán-Fernández, Zorba Blázquez-Bermejo, Beatriz Díaz-Molina, Manuel Gómez-Bueno, Luis Almenar-Bonet, Amador López-Granados, Francisco González-Vílchez, Sonia Mirabet-Pérez, Elena García-Romero, Sobrino-Márquez Jose M., Gregorio Rábago Juan-Aracil, Maria Angels Castel-Lavilla, Teresa Blasco-Peiro, Iris Garrido-Bravo, Luis De La Fuente-Galán, Javier Muñiz, María G. Crespo-Leiro

**Affiliations:** ^1^ Ferrol University Hospital Complex, Ferrol, Spain; ^2^ University Hospital October 12, Madrid, Spain; ^3^ Gregorio Marañón Hospital, Madrid, Spain; ^4^ Central University Hospital of Asturias, Oviedo, Spain; ^5^ Puerta de Hierro University Hospital Majadahonda, Madrid, Spain; ^6^ La Fe Hospital, Valencia, Spain; ^7^ Hospital Universitario Reina Sofía, Cordoba, Spain; ^8^ Marqués de Valdecilla University Hospital, Santander, Spain; ^9^ Hospital de la Santa Creu i Sant Pau, Universitat Autònoma de Barcelona, Barcelona, Spain; ^10^ Bellvitge University Hospital, Barcelona, Spain; ^11^ Virgen del Rocío University Hospital, Seville, Spain; ^12^ University Clinic of Navarra, Pamplona, Spain; ^13^ Hospital Clinic of Barcelona, Barcelona, Spain; ^14^ Hospital Universitario Miguel Servet, Zaragoza, Spain; ^15^ Hospital Universitario Virgen de la Arrixaca, Murcia, Spain; ^16^ Hospital Clínico Universitario de Valladolid, Valladolid, Spain; ^17^ Grupo de Investigación Cardiovascular (GRINCAR), University of A Coruña, A Coruña, Spain; ^18^ A Coruña University Hospital Complex (CHUAC), A Coruña, Spain

**Keywords:** heart transplantation, prognosis, incidence, colorectal cancer, management

## Abstract

In this observational and multicenter study, that included all patients who underwent a heart transplantation (HT) in Spain from 1984 to 2018, we analyzed the incidence, management, and prognosis of colorectal cancer (CRC) after HT. Of 6,244 patients with a HT and a median follow-up of 8.8 years since the procedure, 116 CRC cases (11.5% of noncutaneous solid cancers other than lymphoma registered) were diagnosed, mainly adenocarcinomas, after a mean of 9.3 years post-HT. The incidence of CRC increased with age at HT from 56.6 per 100,000 person-years among under 45 year olds to 436.4 per 100,000 person-years among over 64 year olds. The incidence rates for age-at-diagnosis groups were significantly greater than those estimated for the general Spanish population. Curative surgery, performed for 62 of 74 operable tumors, increased the probability of patient survival since a diagnosis of CRC, from 31.6% to 75.7% at 2 years, and from 15.8% to 48.6% at 5 years, compared to patients with inoperable tumors. Our results suggest that the incidence of CRC among HT patients is greater than in the general population, increasing with age at HT.

## Graphical Abstract

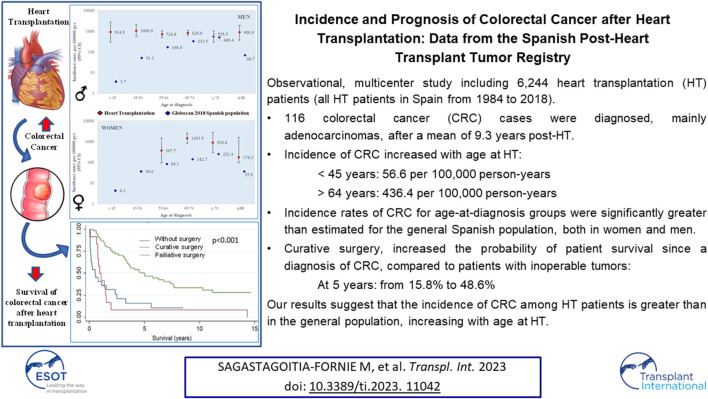

## Introduction

Throughout the last few decades, the life expectancy of patients with a heart transplant (HT) has increased mainly due to advances in immunosuppression, conferring more weight to long-term causes of morbidity–mortality [1], such as malignancies [2, 3]. The risk of *de novo* malignancy in HT recipients was reported to be 2—4 times higher than that in the general population [4–8]. According to the Spanish Post-Heart-Transplant Tumor Registry (SPHTTR), the most common cancer after HT is skin cancer, followed by noncutaneous solid cancers other than lymphoma. Within this latter group, gastrointestinal tumors are the second most frequent, behind lung cancer [9, 10]. Colorectal cancer (CRC) is the third most common malignancy in the general population worldwide, after lung and breast cancers, although it is the most common cancer in Spain [11]. Although CRC seems to increase slightly after transplantation, the representation of HT recipients in most such studies is low but results are controversial [12–14].

The aim of this study was to report on the incidence of CRC (overall and among different subgroups), its characteristics, the treatment received, and survival among HT recipients. This included analyzing SPHTTR data, which is updated yearly with information on tumors in all HT patients since the beginning of this therapy in Spain in 1984, and compare these results with a reference population.

## Materials and Methods

We conducted a retrospective, observational, multicenter study that included all patients who underwent a HT from the beginning of this therapy in Spain in 1984 to the 31st December 2018. As a source of information, we used the SPHTTR, which contains the records of HT patients of Spanish hospitals. From a total of 8,482 patients included in the SPHTTR, we excluded 490 pediatric transplants (<16 years), 1,520 patients who died in the first 3 months after HT, 112 combined transplants, and 116 due to retransplantation. The remaining 6,244 patients were followed up to December 2019.

In order to assess the incidence of CRC in different subgroups, the data considered were sex, age at HT, pre-HT smoking history, obesity, background of CRC pre-HT, immunosuppression treatment, anti-viral prophylaxis received, development of CRC, age at diagnosis of CRC, and duration of follow-up (terminated at the earlier of death or the 31st December 2019). To evaluate the effect of changes in immunosuppressive practice or HT protocols, the era in which the HT was performed was introduced as an independent variable. Two eras were considered: the period before (1984–2000) and after (2001–2018) the introduction of interleukin-2 receptor (IL-2R) blockers in Spain. For characterization of post-HT CRC, additional variables were taken into account such as time between HT and CRC diagnosis, localization of the tumor (colon or rectum), pathological features, extension at diagnosis (metastatic, including lymph nodes, or localized), treatment received (surgery, chemotherapy, radiotherapy), surgical purpose (palliative, curative or none), and survival after CRC diagnosis.

Total incidence (age standardized with the direct method for the world standard population aged >15 years), and incidence in age-at-diagnosis groups (<45, 45–54, 55–64, 65–74, ≥75 years), were compared with GLOBOCAN 2018 estimates for the general Spanish population [15]. A discrepancy between the age of initiation of adulthood used in the SPHTTR (16 years) and the lower limit of the 15–44 years age group used for the world standard population was deemed of no consequence in this study.

This research protocol was approved by the institutional review board of each participating center.

Confidence intervals (CIs) for incidence rates in HT patient age groups were calculated using the quadratic approximation to the Poisson log likelihood for the log rate parameter [16]. Confidence intervals for GLOBOCAN 2018 age‐group–specific incidence rate estimates were calculated using the exact method described by Armitage and Berry [17] with Epidat 4.2 [18]. Confidence intervals for age‐standardized overall incidence rates were calculated as per Fay and Feuer [19, 20] using Epidat 4.2. Adjusted estimates of relative risk were obtained by means of a Poisson regression analysis. A world standard population was used to obtain adjusted rates in HT patients and the Spanish general population [20]. Postdiagnosis survival curves were constructed by a Kaplan-Meier method and compared using log rank tests to estimate the statistical significance of differences.

Except where otherwise stated, all statistical calculations were performed using Stata v12.0.

## Results

### Study Population

This study included 6,244 patients (1,186 women [19.0%] and 5,058 men [81.0%]) with a total follow-up of 55396.5 person-years (pys), median follow-up of 8.8 years, and a mean age at HT of 52.1 years. A total of 976 (21.0%) patients were smokers pre-HT, 683 (11.3%) obese, and 16 (7.4%) had a history of CRC before HT surgery. A total of 2,553 patients (40.9%) underwent HT in the pre-IL2R–blocker era, and 3,691 (59.1%) in the most recent era. Of the total patients, 83.0% received induction therapy and 60.6% antivirals post-HT (Table 1). The used immunosuppressive agents are listed in (Table 2).

**TABLE 1:** Patient characteristics.

Number of patients	6,244
Female	1,186 (19.0%)
Mean (SD) age at HT	52.1 (11.5)
Age at HT	
<45 years	1,340 (21.5%)
45–54 years	1,759 (28.2%)
55–64 years	2,516 (40.3%)
≥65 years	629 (10.0%)
Pre-HT smoking^a^	976 (21.0%)
Obesity^b^	683 (11.3%)
Pre-HT colon or rectum tumor^c^	16 (7.4%)
HT era	
1984–2000	2,553 (40.9%)
2001–2018	3,691 (59.1%)
Induction therapy	4,992 (83.0%)
Aciclovir or Ganciclovir after HT^d^	3,387 (60.6%)

^a^ Out of 4,653 patients for whom relevant data were available.

^b^ Out of 6,048 patients for whom relevant data were available.

^c^ Out of 215 patients for whom relevant data were available.

^d^ Out of 5,586 patients for whom relevant data were available.

**TABLE 2:** Patients receiving each kind of immunosuppressor (percentages), by period post-HT.

	<3 months	3–12 months	1–2 years	After 2 years	At any time
Cyclosporine	58.2	52.8	46.5	38.5	59.1
Azathioprine	36.2	32.4	27.7	19.4	37.2
Prednisone	85.6	76.6	56.4	44.4	86.0
Tacrolimus	30.1	26.5	19.5	18.3	39.3
MMF	49.7	42.3	34.0	35.4	62.6
Sirolimus	0.7	0.8	0.9	3.8	4.5
Everolimus	1.5	2.6	3.3	7.0	9.7
OKT3	23.4	0.2	0.1	0.1	23.6
Anti-thymocyte globulin	5.6	0.8	0.2	0.1	5.6
Basiliximab	27.7	0.1	0.0	0.0	27.7
Daclizumab	7.0	0.2	0.0	0.0	7.0
N = 6,242					

### Incidence of Colorectal Cancer After Heart Transplantation

With regard to tumors, 2,498 cases were registered, of which 116 were CRC (4.6% of all tumors and 11.5% of noncutaneous solid cancers other than lymphoma). Of these 116 cases, 99 were diagnosed in men and 17 in women. The incidence of CRC increased with age at HT from an average of 56.6 per 100,000 pys among under 45 year olds to 436.4 per 100,000 pys among over 64 year olds. No statistically significant differences were observed related to sex, pre-HT smoking history, obesity, HT era, immunosuppressive practice or antiviral prophylaxis (Table 3).

**TABLE 3:** Incidence of colorectal tumors per 100,000 person-years among heart transplant patients and different subgroups. Follow-up, cases, incidence rates and relative risk.

Group	Follow-up (Pys)	Cases	Incidence rate	95% CI		RR	95% CI		p
Total	55396.5	116	209.4	174.6	251.2				
Sex									
Male	44763.6	99	221.2	181.6	269.3	1			
Female	10632.9	17	159.9	99.4	257.2	0.7	0.4	1.2	0.215
Age at HT (years)									
<45	14147.8	8	56.6	28.3	113.1	1			
45–54	16485.9	32	194.1	137.3	274.5	3.4	1.6	7.5	0.002
55–64	20638.3	58	281.0	217.3	363.5	5.0	2.3	10.4	<0.001
≥65	4124.5	18	436.4	275.0	692.7	7.7	3.4	17.8	<0.001
Pre-HT smoking	8721.0	13	149.1	8.6	256.7	0.6	0.4	1.1	0.124
Obesity	5520.2	9	163.0	84.8	313.3	0.8	0.4	1.5	0.471
Pre-HT colorectal tumor	115.9	2	1726.3	431.7	6902.5	8.3	2.1	33.5	<0.001
HT era									
1984–2000	28797.4	56	194.5	149.7	252.7	1			
2001–2018	26599.1	60	225.6	175.1	290.5	1.2	0.8	1.7	0.424
Immunosuppression									
OKT3 (Yes/No)	16864.4	44	260.9	194.2	350.6	1.4	0.9	2.0	0.104
ATG (Yes/no)	6530.4	14	214.4	127.0	362.0	1.0	0.6	1.8	0.982
Basiliximab (Yes/No)	14547.9	25	171.9	116.1	254.3	0.8	0.5	1.2	0.205
Daclizumab (Yes/no)	4096.0	11	268.6	148.7	484.9	1.3	0.7	2.4	0.424
Antiviral prophylaxis									
Aciclovir (Yes/No)	20118.5	42	208.8	154.3	282.5	0.9	0.7	1.4	0.788
Ganciclovir (Yes/No)	18822.8	39	207.2	151.4	283.6	0.9	0.6	1.4	0.768
Aciclovir or Ganciclovir	30373.0	67	220.6	173.6	280.2	1.1	0.73	1.56	0.734

The mean age at diagnosis was 66.0 years (SD 8.8), with three patients under 45, nine aged 45–54 years, 29 aged 55–64 years, 62 aged 65–74 years, and 13 ≥ 75 years.

### Incidence Comparison With the General Spanish Population

**FIGURE 1:**
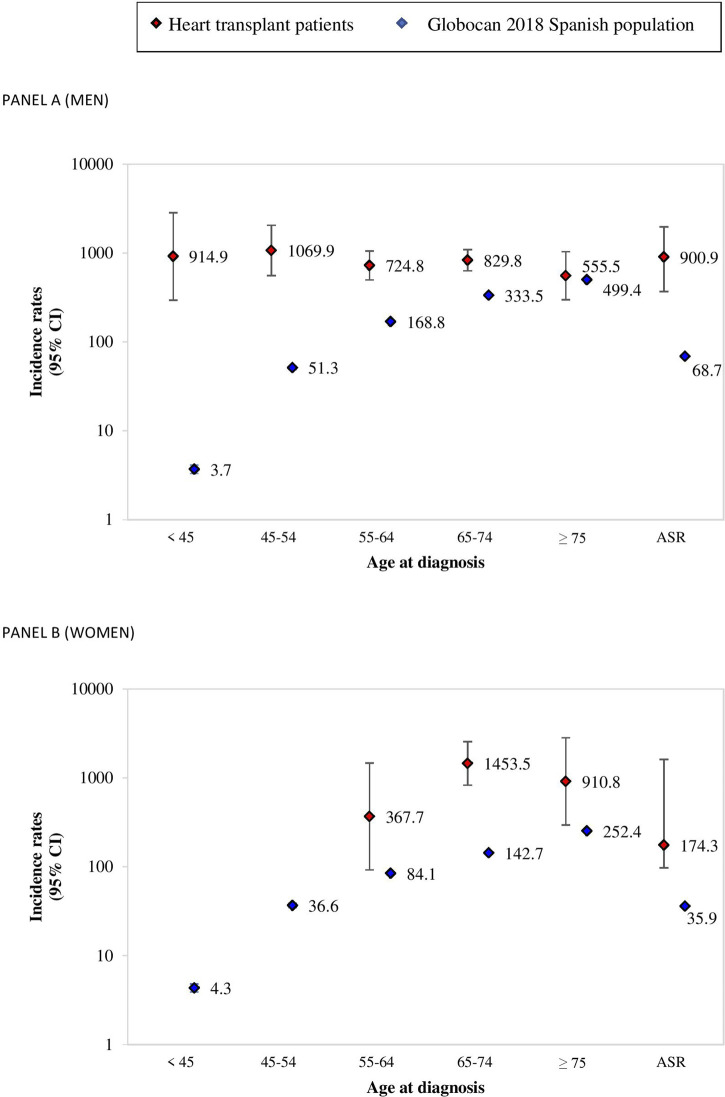
Incidence rates per 100,000 person-years for age-at-diagnosis groups, and age-standardized overall rates for colorectal cancer among HT patients (SPHTTR data) and for the general Spanish population (GLOBOCAN 2018). **(A)** men; **(B)** women.

Contrary to what is observed in the general population, the incidence of CRC post-HT remained fairly constant with increasing age, particularly among males. However, both age- and sex-specific CRC incidences as well as age-standardized overall rates were several-fold higher in every group than GLOBOCAN 2018 estimates for the Spanish population, except for the subgroup of men over 75 for whom no statistically significant differences were found (Figure 1).

### Colorectal Cancer Characteristics After Heart Transplantation

The mean time between HT and diagnosis of CRC was 9.3 years (SD 5.5 years). Information on tumor extension status at diagnosis was available in 107 cases, the cancer being metastatic in only 12 patients (11.3%). Regarding histopathological characteristics, 91 (85.1%) were adenocarcinomas (Table 4). We had no information on the treatment received by 18 patients. Curative surgery was performed on 62 patients (63.3%) and palliative surgery on 12 (12.2%), whereas 24 patients (24.5%) did not undergo any surgical treatment. Chemotherapy was indicated in 32 cases (33.7%) and radiotherapy in 13 (13.7%).

**TABLE 4:** Characteristics of colorectal tumors at diagnosis (results expressed as n (%) unless otherwise stated).

Number of colorectal tumors	116
Mean (SD) age (years) at diagnosis	66.0 (8.8)
Mean (SD) time (years) since HT	9.3 (5.5)
Location	
Colon	92 (79.3)
Rectum	22 (19.0)
Anal	2 (1.7)
Histology^a^	107
Adenocarcinoma	81 (75.7)
Metastatic adenocarcinoma	10 (9.4)
Carcinoma	7 (6.5)
Metastatic carcinoma	2 (1.9)
Epidermoid carcinoma	3 (2.8)
Lynphoproliferative Syndromes	4 (3.7)
Surgery^b^	98
None	24 (24.5)
Paliative	12 (12.2)
Curative	62 (63.3)
Radiotherapy^c^	13 (13.7)
Chemotherapy^c^	32 (33.7)
Response to treatment^d^	87
Complete	50 (57.5)
Partial	17 (19.5)
None	20 (23.0)
Aciclovir or Ganciclovir	2 (1.7)

^a^ Out of 107 patients for whom relevant data were available.

^b^ Out of 98 patients for whom relevant data were available.

^c^ Out of 95 patients for whom relevant data were available.

^d^ Out of 87 patients for whom relevant data were available.

### Prognostic Impact of Colorectal Cancer After Heart Transplantation

**FIGURE 2:**
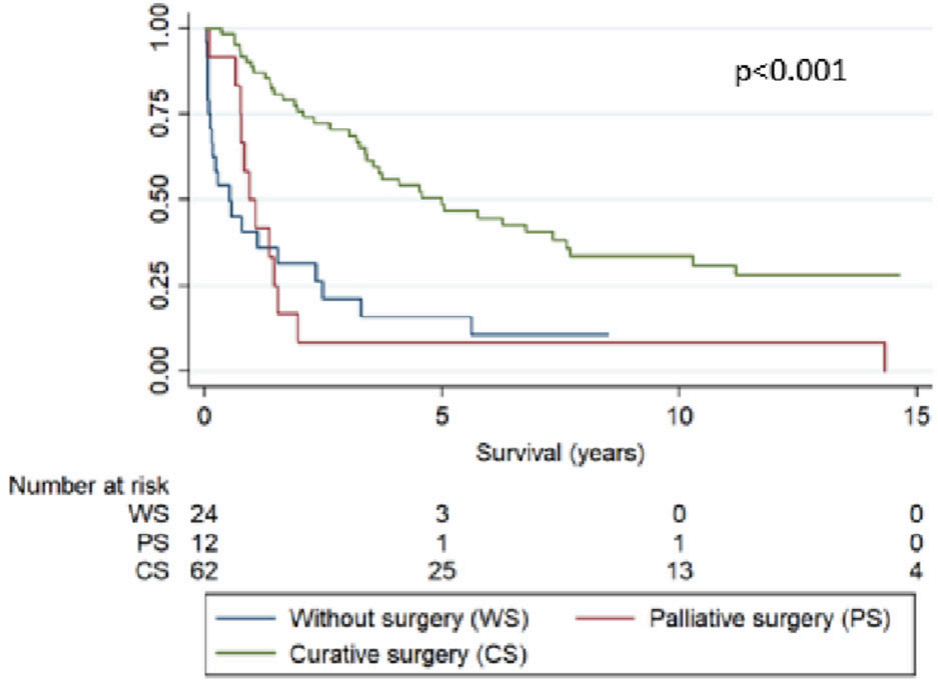
Survival curves since diagnosis of colorectal cancer after HT by type of surgical treatment (*p* < 0.001).

Within 2 and 5 years after diagnosis, overall Kaplan–Meier survival fell to 59.1% and 39.1%, respectively. No statistically significant differences were observed in the survival curves related to sex (women vs. men), nor to age at diagnosis (under vs. over 55). Curative surgery, performed in 62 of the 74 operable cases, increased the probability of survival since diagnosis from 31.6% to 75.7% at 2 years, and from 15.8% to 48.6% at 5 years, compared to inoperable patients (Figure 2).

## Discussion

### Incidence of Colorectal Cancer After Heart Transplantation

In this study, we found the incidence of CRC considerably greater among Spanish HT patients than that which corresponded to the GLOBOCAN 2018 estimate for the general Spanish population.

Solid organ transplant recipients are at increased risk of cancer [3]. Regarding HT in particular, Youn et al. [4] found that more than 10% of adult HT recipients from the International Society for Heart and Lung Transplantation (ISHLT) registry developed *de novo* malignancy between years 1 and 5 after transplantation, and this outcome was associated with greater mortality. The largest increase was detected in skin cancer, followed by noncutaneous solid cancer, even though, more specifically, the proportion of CRC cases was lower than in our cohort. This result might be explained by the mean time between HT and the diagnosis of CRC in our study being over 9 years, whereas the ISHLT registry included only tumors within 5 years post-HT. The incidence rate of CRC after HT in our analysis was 209.4 per 100,000 pys (4.6% of the total malignancies); Van Keer et al. [21] reported similar numbers in Leuven.

The incidence of CRC increased with age at HT, which was expected, given the known relationship between age and cancer, but surprisingly this trend was not observed with age at diagnosis without a clear justification. Our group [22] had already noticed that the incidence rate ratio of lung cancer between HT and general populations fell with increasing age, suggesting, as a possible explanation, that with aging the effect of immunosuppression is relatively reduced due to a gradual decline of the immune system.

### Incidence Comparison With a Reference Population

Furthermore, the data reported in most studies are incidence rates that are not normalized to the general population. The SPHTTR data analyzed in our study showed an increased incidence of CRC in patients undergoing HT compared to GLOBOCAN 2018 estimates for the general population in Spain. This difference was maintained in successive age-at-diagnosis groups and age-standardized overall rates, except for the subgroup of men over 75 for whom although a rising trend was observed it was not statistically significant. In addition, the incidence of CRC has been increasing over the past few years, being at present the commonest tumor in Spain [11]. Therefore, to compare our cohort, which includes patients since 1984, to a general population obtained from GLOBOCAN 2018 might seem conservative but, nevertheless, the incidence post-HT was found to be higher than in the general population. Despite GLOBOCAN estimates that may possibly lead to an error owing to finite sampling, the aforementioned point, together with observed differences, suggest a greater risk of CRC after HT, which adds consistency to our results. Jäämaa-Holmberg et al. [23] recently assessed cancer incidence and mortality in Finnish HT recipients, both being markedly increased after HT, in comparison to the general Finnish population. Regarding CRC, they reached similar conclusions, observing that the incidence of colon cancer was 3.5–4 times higher than in the general Finnish population, although no rectal cancer was registered in their post-HT records. In contrast, Kellerman et al. [8] described no significant differences in relation to the incidence of CRC among the United States (US) HT and general populations. A possible explanation could be that the reference population considered in this US study might have been unsuitable for their HT cohort.

### Potential Risk Factors Associated With Colorectal Cancer

Risk factors, such as obesity or smoking, have been associated with the development of CRC in the general population [24, 25], yet no statistically significant differences were observed in our analysis. Moreover, in spite of the fact that in our study the proportions of smokers and obese patients were lower than those of non-smokers and non-obese patients, respectively, the risk of CRC was still higher. These findings, together, suggest that the increased incidence is not due to these modifiable factors. Conversely, as was expected, even though the subgroup with a background of CRC pre-HT was quite small, the likelihood of this developing CRC after HT was eight times greater.

The association between immunosuppression and increased risk of neoplasia is well known. However, it is difficult to identify which immunosuppressive regimens are associated with an increased risk of cancers. No statistically significant differences were observed related to HT era or immunosuppressive agents in our study. Given a lack of information in the literature on the effect of immunosuppression specifically on CRC after HT, we can only compare such data with outcomes of malignancy in more general subgroups such as patients with noncutaneous solid tumors. In this subgroup, in terms of induction therapy, OKT3 and anti-thymocyte globulin have been associated with an increase in cancers [5], whereas in the Youn et al. analysis [4], mycophenolate mofetil, which is known to have anti-proliferative properties and prevent tumor dissemination by inhibiting endothelial cell proliferation and angiogenesis [25], showed a protective effect compared to azathioprine.

### Colorectal Cancer Characteristics After Heart Transplantation

Regarding the characterization of post-HT colorectal cancer, consistent with CRC in general, the main histopathological type was adenocarcinoma. Furthermore, in our HT cohort most CRC cases were not metastatic at diagnosis, possibly due the close follow-up HT patients undergo, even though recommendations regarding CRC screening in HT recipients do not differ from those of the general population [26], which in Spain consists in fecal occult blood test beginning at age 50, followed by endoscopic study if positive. However, even though only 11.2% of cases were metastatic at diagnosis, surprisingly almost 25% were not considered operable. We do not know if that decision was due to high surgical risk or another reason. Curative surgery was performed in 63% of cases, increasing the probability of survival.

### Limitations

The limitations of our study ought to be taken into account when interpreting the reported results. First, the study was retrospective. Second, because no Spanish national cancer registry existed, the incidence of CRC in the general population was obtained from GLOBOCAN 2018 estimates. However, as CRC incidence is increasing, comparing our data related to HT since 1984 to a 2018 reference population might underestimate the difference in incidences between both cohorts, strengthening our results. Third, the SPHTTR may have been missing some tumors because, while we do assure that every cancer included in our records is verified, some cases might not have been detected if diagnosed in a referral center where neither the patient nor their physician informed their corresponding transplant unit. Nevertheless, this would again underestimate the incidence of CRC in our HT population and, therefore, strengthen our results. Finally, although all Spanish hospitals performing HT in adults continually update data in the SPHTTR, it does not contain information that might have been of interest to analyze, such as CRC location (right or left sided), TNM staging, more specific details related to treatment, the implementation or not of CRC screening tests (fecal occult blood test or colonoscopy) in pre-HT protocols, and the use of statins [27] and aspirin [28] since some studies suggest these drugs might have a protective effect against CRC.

### Conclusion

We conclude that the incidence of CRC among HT patients is greater than in the general population in our country, increasing with age at HT. Curative surgery increased the probability of survival compared to palliative surgery or that of inoperable patients. This suggests that a post-cardiac transplant follow-up might require specific screening for this cancer to achieve early diagnosis and treatment since this improves health outcomes, particularly after a recent recommendation to move the age at which to start screening for colorectal cancer in the general population forward from 50 to 45 years [29].

## Data Availability

The raw data supporting the conclusion of this article will be made available by the authors, without undue reservation.

## Abbreviations

CRC, colorectal cancer; CIs, confidence intervals; HT, heart transplantation; ISHLT, International society for heart and lung transplantation; Pys, person-years; SPHTTR, Spanish post-heart-transplant tumor registry.
